# Central modulation of cardiac baroreflex moment‐to‐moment sensitivity during treadmill exercise in conscious cats

**DOI:** 10.14814/phy2.15371

**Published:** 2022-06-27

**Authors:** Kei Ishii, Mitsuhiro Idesako, Ryota Asahara, Nan Liang, Kanji Matsukawa

**Affiliations:** ^1^ Human Informatics and Interaction Research Institute National Institute of Advanced Industrial Science and Technology Ibaraki Japan; ^2^ Department of Integrative Physiology, Graduate School of Biomedical and Health Sciences Hiroshima University Hiroshima Japan; ^3^ Cognitive Motor Neuroscience, Human Health Sciences Graduate School of Medicine, Kyoto University Kyoto Japan

**Keywords:** baroreflex slope, central command, exercise intensity transition, voluntary exercise

## Abstract

It remains undetermined whether the cardiac component of the entire arterial baroreflex is blunted even at the onset of low‐intensity exercise. We sought to examine the moment‐to‐moment sensitivity of the cardiac baroreflex during walking at different speeds and the presumed mechanisms responsible for baroreflex modulation in conscious cats. Arterial baroreflex sensitivity for heart rate was estimated from the baroreflex ratio between changes in systolic arterial blood pressure and heart rate and from the slope of the baroreflex curve between the cardiovascular responses to brief occlusion of the abdominal aorta. Treadmill walking was performed for 1 min at three levels of speed (low: 20–30 m/min, moderate: 40 m/min, and high: 50–60 m/min) or for 3 min at the stepwise change of speed (low to high to low transition). Cardiac baroreflex sensitivity was blunted at the onset of walking, irrespective of speed. Thereafter, the blunted cardiac baroreflex sensitivity was restored around 15 s of walking at any speed, while the blunting occurred again at 45 s of high‐speed walking. The inhibition of cardiac baroreflex sensitivity also occurred (1) during the speed transition from low to high and (2) at 45 s of high‐speed exercise of the stepwise exercise. The blunted cardiac baroreflex sensitivity was restored immediately to the resting level during the speed transition from high to low, despite sustained pressor and tachycardiac responses. Therefore, moment‐to‐moment modulation of the cardiac baroreflex during exercise would occur in association with motor intention (i.e., exercise onset) and effort (i.e., treadmill speed).

## Graphical abstract

The cardiac component of the arterial baroreflex was blunted even at the onset of low‐intensity exercise and during the transition of exercise intensity from low to high, but the blunting stops immediately during the transition from high to low intensity. The moment‐to‐moment modulation of the cardiac baroreflex during exercise would be driven by central command.

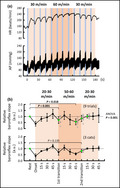

## 1 INTRODUCTION

The arterial baroreflex (i.e., the aortic and carotid sinus baroreflexes) maintains arterial blood pressure (AP) at a constant level by rapid regulation of cardiac output (chiefly heart rate [HR]), being followed by peripheral vascular regulation via the autonomic nervous system (Fadel & Raven, 2012; Joyner, 2006; Matsukawa, 2012; Miki & Yoshimoto, 2018; Rowell & O'Leary, 1990; Sagawa, 1983). The cardiac component of the arterial baroreflex plays an important role in the rapid buffering of AP changes (Ogoh et al., 2003). The feedback mechanism continues to function not only at rest but also during exercise (Krasney et al., 1974; Krieger et al., 1998; Ludbrook & Graham, 1985; Sadamoto & Matsukawa, 1997; Walgenbach & Donald, 1983). However, when cardiac acceleration is required to rapidly increase cardiac output, such as transitions from rest to exercise or from low‐ to high‐intensity exercise, the cardiac component of the arterial baroreflex should be suppressed to maintain adequate cardiac output for ensuring blood flow to the required organs such as contracting skeletal muscles and the brain.

Our laboratory has previously examined the dynamic modulation of cardiac baroreflex function using brief electrical stimulation of baroafferent nerves or a mechanically induced rise in AP in cats (Komine et al., 2003; Matsukawa et al., 2012; Matsukawa et al., 2013; Matsukawa et al., 2015; Matsukawa et al., 2016; Murata et al., 2004). It has been demonstrated that the baroreflex control of HR is blunted at the onset of voluntary static exercise in conscious cats and spontaneous motor activity in decerebrate cats. Later, Fisher et al. (2007) and Barbosa et al. (2016) reported in humans that the cardiac component of carotid sinus baroreflex was blunted at the onset of isometric handgrip and during the first 35 s of dynamic cycling when exercise intensity was moderate or high, but not low. These results suggest that exercise intensity is a key factor in the suppression of the cardiac component of carotid sinus baroreflex at the onset of exercise. However, the aortic baroreflex plays a superior role in HR control, not only in animals (Ishii et al., 2015; Pickering et al., 2008) but also in humans (Ferguson et al., 1985; Shi et al., 1993; Smith et al., 2001). In decerebrate cats, the inhibition of cardiac baroreflex sensitivity at the onset of spontaneous motor activity resulted chiefly from inhibition of the cardiac component of aortic baroreflex, but not carotid sinus baroreflex (Matsukawa et al., 2012; Matsukawa et al., 2013). This background information led us to hypothesize that the cardiac component of the entire arterial baroreflex (i.e., both aortic and carotid sinus baroreflexes) is blunted even at the onset of low‐intensity exercise in conscious animals and humans. Verification of this hypothesis and the responsible mechanism(s) is important, considering the relationship between cardiac acceleration at the onset of exercise and adverse cardiac events in patients with coronary artery disease (Falcone et al., 2005).

The most plausible mechanism responsible for the blunting of cardiac baroreflex sensitivity at the onset of exercise is a central command (i.e., feedforward signal descending from higher brain centers) rather than exercise pressor reflex (i.e., feedback signal via mechano‐ and metabosensitive afferents in contracting skeletal muscles) (Komine et al., 2003; Matsukawa et al., 2012; Matsukawa et al., 2013; Matsukawa et al., 2015; Matsukawa et al., 2016; Murata et al., 2004), while either mechanism would contribute to resetting of the baroreflex during exercise (Gallagher et al., 2001; McIlveen et al., 2001; Potts & Mitchell, 1998; Smith et al., 2003). If central command operates to blunt the cardiac baroreflex, such baroreflex modulation will occur depending on the characteristics of the central drive in association with motor intention and/or effort (Gandevia et al., 1993; Green & Paterson, 2008; Matsukawa, 2012; Mitchell & Victor, 1996; Williamson et al., 2006), that is, baroreflex bradycardia in response to a rise in AP would be blunted during the transition from rest to exercise or from low to high effort and during high‐intensity exercise. On the other hand, the blunted baroreflex bradycardia during high‐intensity exercise would be immediately restored during the transition from high‐ to low‐intensity exercise. However, such central command‐like dynamic modulation of the cardiac baroreflex remains undetermined.

Along this line, we hypothesized that central command blunts the cardiac component of the arterial baroreflex even at the onset of low‐intensity exercise and during the transition of exercise intensity from low to high, but the blunting stops immediately during the transition from high to low intensity. To test the hypothesis, this study examined (1) the effect of relatively low‐intensity exercise on the cardiac component of arterial baroreflex and (2) the cardiac baroreflex sensitivity during transitions of exercise intensity in conscious cats. Cardiac baroreflex function was assessed, with brief mechanical occlusion of the abdominal aorta, during treadmill walking at three different levels of speed (i.e., exercise intensity) or stepwise change of speed (i.e., transition of intensity).

## 2 METHODS

### 2.1 Ethics statement

The present study was conducted in three female cats (age, >2 years at the start of training; weight, 2.7–3.5 kg) in accordance with the “Guiding Principles for the Care and Use of Animals in the Fields of Physiological Sciences” approved by the Physiological Society of Japan and the Institutional Animal Experimental Committee, Hiroshima University Faculty of Medicine. The experimental protocols were submitted to and approved by the Committee of Research Facilities for Laboratory Animal Science, Natural Science Center for Basic Research and Development, Hiroshima University (A13‐75). All experiments complied with the ARRIVE guidelines 2.0 (Percie du Sert et al., 2020) and principles of animal research (Grundy, 2015) and were performed at Hiroshima University. The cats were mongrel and bred (Nagoya Laboratory, Nagoya, Japan). They were housed and fed once a day in individual cages in a temperature‐controlled room (≈25°C).

### 2.2 Training and implantation surgery

The three cats were selected because they did not appear to hesitate to (1) rest on a treadmill, (2) perform treadmill exercise, and (3) wear a fitted jacket. The cats were trained over at least 5 weeks (4 days/week) to accustom them to running on a motor‐driven treadmill as previously reported (Tsuchimochi et al., 2002). The training session consisted of five exercise bouts for 1–3 min at the running speed of 10–60 m/min at 0% grade.

After the training period, the cats were initially anesthetized by inhalation of a 3–4% halothane–N_2_O–O_2_ gas mixture in order to implant recording electrodes and catheters. Each cat was intubated with an endotracheal tube. HR, rectal temperature, and thoracic respiratory movement were continuously monitored during the surgery. To maintain an appropriate level of surgical anesthesia, the concentration of halothane was increased in the range of 1.0–2.5% if a noxious pinch of the paw or a surgical procedure increased HR and/or respiration or caused the withdrawal of the limb. Rectal temperature was maintained at 37–38°C with a heating pad. Polyurethane catheters were inserted into the right cephalic vein or left external jugular vein for administration of drugs and into the right brachial artery for measurement of AP. Thereafter, the abdominal aorta was retroperitoneally exposed and carefully isolated using an operating microscope. An inflatable cuff occluder (VO‐4, Unique Medical Co., Japan) filled with sterilized saline was wrapped around the abdominal aorta to mechanically evoke a brief increase in AP (i.e., activate both aortic and carotid sinus baroreceptors) by inflating the occluder. In one cat, a pair of polytetrafluoroethylene (Teflon)‐coated silver wire electrodes were inserted into the biceps brachialis muscles of bilateral forelimbs for recording muscle activities. The original electromyogram (EMG) signal was amplified with a band‐pass filter of 50–3000 Hz. The catheters and lead wires were tunneled subcutaneously, brought to the exterior in the intrascapular region, and then protected with a fitted jacket.

After the implantation surgery, antibiotics (20,000 U/kg intramuscular benzylpenicillin potassium) were administered, and the cats were housed in their cages and warmed with a heating pad and an external lamp. Antibiotics (100,000 units benzylpenicillin benzathine, Bicillin Tablets, Banyu Pharmaceutical, Tokyo, Japan) were also orally administered for 5–7 postoperative days. Arterial and venous catheters were flushed and filled with heparinized saline every day. The cats were able to stand up, walk, and eat food on the day after the surgery. After 1 week postoperatively, HR and AP in the resting state returned to presurgical levels and the cats seemed to be comfortable and voluntarily ran overground.

### 2.3 Experimental protocols

The cats seemed to be in good condition to run on the motor‐driven treadmill by postoperative days 8–14. This period seems to be sufficient for the cats to have recovered enough to run as previously reported (Desrochers et al., 2019). The cats were repeatedly examined 3–4 times/week over 1–2 months. On the day of the experiment, each cat was put into a transparent plastic box (size, 65 × 20 × 35 cm) that was placed on the treadmill. The arterial catheter and EMG wires were connected to a pressure transducer and a light lead cable, respectively. The aortic occluder was connected to a 1.0 ml syringe via a sterilized saline‐filled extension tube.

To examine the effect of exercise intensity on arterial baroreflex control of HR during exercise, brief occlusion (≈3 s) of the abdominal aorta by inflation of the occluder was induced at rest, around the onset of exercise (<5 s from exercise onset), and during treadmill exercise (≈15, 30, and 45 s from exercise onset). When the cats stood on four legs quietly and the cardiovascular variables became stable, the treadmill exercise (0° incline) was started and performed for 1 min at three different levels of speeds (low: 20–30 m/min, moderate: 40 m/min, and high: 50–60 m/min), ranging from a slow walk to brisk trot (Hoffer et al., 1987; Kaya et al., 2003). Similarly, the cats performed treadmill exercises without aortic occlusion to calculate the net decrease in HR (ΔHR_net_) caused by aortic occlusion (as described below). It took 3–5 s for the treadmill speed to reach a steady‐state level from zero. After treadmill exercise, a bite‐sized meal was occasionally given to the cats as a reward. Each treadmill exercise was followed by 10–20 min of rest, depending on the treadmill speed.

The central command operates in association with motor intention and effort, which in turn would blunt the baroreflex control of HR (Matsukawa, 2012; Mitchell & Victor, 1996; Williamson et al., 2006). This concept highlights the possibility that baroreflex control of HR is blunted by central command, which is driven by increasing exercise speed (i.e., increasing motor effort), whereas decreasing exercise speed (i.e., decreasing motor effort) reduces or stops driving central command and thereby does not blunt the baroreflex control of HR. To test this possibility, the cats performed treadmill exercises with stepwise (convex) changes in speed (20–30 to 50–60 to 20–30 m/min). Each speed was maintained for 1 min. Aortic occlusion was induced every 15 s.

Although we attempted to randomize the order of treadmill exercise as much as possible, the experiments were frequently started with low‐speed exercise to confirm the cat's condition on the day of the experiment. When the cats seemed to hesitate or did not perform treadmill exercises, the experiment was discontinued. After the end of the experiments, the cats were used in other decerebrate experiments at Hiroshima University and received a lethal dose of pentobarbital sodium through the cephalic vein.

### 2.4 Recording of data

Timings at the onset and end of treadmill exercise (i.e., when the cats began to walk and when they stopped walking) were determined by using a manually and electrically marked signal or EMG signal. Similarly, timings of occlusion were marked. The marking signals, AP, and EMG were simultaneously recorded on an eight‐channel pen‐writing recorder (Recti‐8 K, GE Marquette Medical Systems, Tokyo, Japan) and stored in a computer with an analog‐to‐digital converter (MP100, BIOPAC Systems, Santa Barbara, CA, USA) at a sampling frequency of 1 kHz. HR was estimated from the AP wave. The beat‐to‐beat values of HR and systolic, mean, and diastolic AP (SAP, MAP, and DAP, respectively) were recalculated using a software program (AcqKnowledge 3.9.1, BIOPAC Systems, Santa Barbara, CA, USA).

### 2.5 Data treatment and statistical analysis

Some trials (29 out of 94 walking trials) and data (17 out of 328 occlusions in the remaining 65 trials) were excluded due to signal artifacts caused by head and body shaking during the treadmill exercise or inadequate pressor responses (ΔSAP < 10 mmHg) to aortic occlusion. Baseline HR and AP were defined as the means of values obtained at rest, 10 heartbeats before aortic occlusion (Table 1). Cardiac baroreflex function at rest and during exercise was analyzed using beat‐by‐beat data of HR and AP in response to aortic occlusion as follows. First, as previously reported (Matsukawa et al., 2012; Matsukawa et al., 2013; Matsukawa et al., 2015; Murata et al., 2004), ΔHR_net_ caused by aortic occlusion was calculated as ΔHR_net_ = ΔHR_ref_ − ΔHR_occl_. ΔHR_occl_ is the peak change in HR, in response to aortic occlusion, from the mean value obtained 10 beats preceding occlusion. ΔHR_ref_ is the mean change in HR at the time when ΔHR_occl_ was detected during treadmill exercise without aortic occlusion. The baroreflex ratio between ΔHR_net_ and increase in SAP or MAP was calculated at rest and during treadmill exercise. The relative baroreflex ratio during exercise was also calculated against the mean ratio at rest in each cat. As another measure to estimate cardiac baroreflex function, changes in HR, SAP, and MAP in response to aortic occlusion were used to construct the arterial baroreflex curves between HR and SAP or MAP for every 5 mmHg increase. These plots were made taking into account a delay of 2–6 beats from occlusion‐induced pressor response to baroreflex bradycardia as previously reported (Matsukawa et al., 2012; Murata et al., 2004). The baroreflex sensitivity for HR was estimated from the maximal slope of the baroreflex curves (Matsukawa et al., 2012; Matsukawa et al., 2013; Matsukawa et al., 2015; Matsukawa et al., 2016). The relative baroreflex slope during exercise was also calculated against the mean slope at rest in each cat.

**TABLE 1:** Baseline values of cardiovascular variables

	Cat A	Cat B	Cat C
HR (beats/min)	179 ± 10	174 ± 5	149 ± 6
SAP (mmHg)	147 ± 7	130 ± 6	111 ± 9
MAP (mmHg)	122 ± 6	112 ± 4	93 ± 7
DAP (mmHg)	97 ± 5	91 ± 4	74 ± 6
Baroreflex slope (beats/min/mmHg)	−2.12 ± 0.78	−1.38 ± 0.68	−0.69 ± 0.22
*n* (trials)	19	19	26

*Note*: Baseline values were defined as the mean values obtained 10 beats before aortic occlusion at rest. Baroreflex slope was calculated from the relationship between SAP and HR changes in response to aortic occlusion at rest. Values are means ± SD.

Abbreviations: DAP, diastolic arterial blood pressure; HR, heart rate; MAP, mean arterial blood pressure; SAP, systolic arterial blood pressure; SD, standard deviation.

Regarding the baroreflex ratio and slope, the following statistical analyses were conducted only in SAP‐related values because the ΔSAP‐ΔHR curve seemed to include saturation point (i.e., lower plateau) (Matsukawa et al., 2013), and a similar blunting effect of cardiac baroreflex was observed, irrespective of using SAP or MAP. All variables were summarized in the number of trials as previously reported (Matsukawa et al., 2012; Matsukawa et al., 2013; Matsukawa et al., 2014; Matsukawa et al., 2015; Matsukawa et al., 2016). We considered that relative values of baroreflex slope and ratio were more appropriate than absolute values because the degree of baroreflex bradycardia in response to aortic occlusion varied among the cats (Table 1). Therefore, statistical results for relative values are described in “Results.” The relative baroreflex ratio and slope were also averaged over trials in each cat and further averaged among the three cats.

Regarding treadmill exercise without occlusion, the preexercise values of cardiovascular variables were defined as mean values obtained over 30 s before the onset of treadmill exercise. Cardiovascular changes from the preexercise values were aligned in each trial based on the timing signal and averaged every 1 s. The cardiovascular responses were further averaged around the onset (1–5 s) and during the late period (51–60 s) of treadmill exercise.

After normality and equal variance tests were performed, the relative baroreflex ratio and slope were analyzed with a one‐way analysis of variance (ANOVA) or Kruskal–Wallis one‐way ANOVA on ranks test (to assess the effect of exercise time or treadmill speed). Post hoc test was performed with Holm–Sidak or Dunn's method. To further confirm the effect of exercise intensity or speed transition on cardiac baroreflex sensitivity, the averaged relative baroreflex slope and ratio were calculated for the three cats, and the values at rest were compared with the values at the onset of treadmill exercise or during the speed transition from high to low by using a two‐tailed paired *t* test (instead of one‐way repeated measures ANOVA, due to the small number of cats). The level of significance was set at *p* < 0.05. All statistical analyses were conducted using SigmaPlot, version 14.0 (Systat Software, San Jose, CA, USA). All variables are expressed as mean ± standard deviations unless otherwise stated.

## 3 RESULTS

### 3.1 Cardiovascular responses to treadmill exercise without aortic occlusion

**FIGURE 1:**
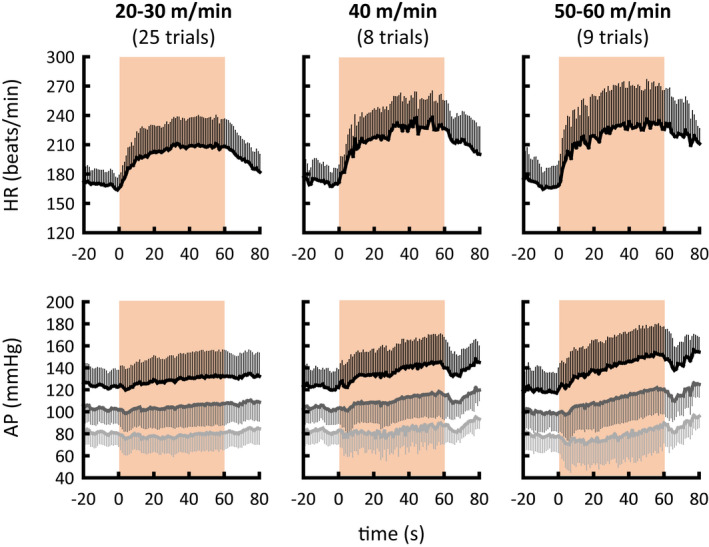
Time courses of HR and AP during treadmill exercise at three levels of speed. Cats performed treadmill exercises for 1 min (orange areas) each at speeds of 20–30, 40, and 50–60 m/min. In the lower panels, black, dark gray, and light gray indicate systolic, mean, and diastolic AP, respectively. Values are means ± standard deviations. AP, arterial blood pressure; HR, heart rate.

**FIGURE 2:**
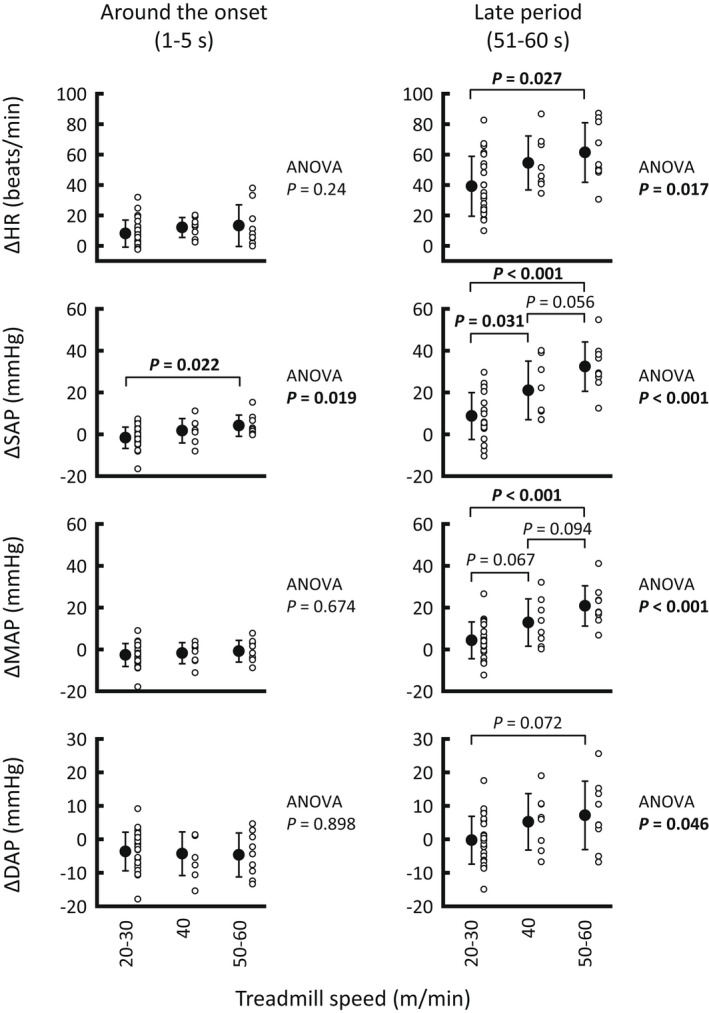
Averaged HR and AP responses around the onset (1–5 s) and during the late period (51–60 s) of treadmill exercise at three levels of speed. Cardiovascular responses, except systolic AP, were independent of treadmill speed around the onset of exercise, and they were augmented in proportion to the speed during the late period of exercise. The number of trials was 25 at low speed (20–30 m/min), 8 at moderate speed (40 m/min), and 9 at high speed (50–60 m/min). Open circles indicate individual values. Filled circles with error bars indicate means ± standard deviations. Each *p* value for post hoc tests is described when *p* < 0.1. *p* < 0.05 are in bold. AP, arterial blood pressure; DAP, diastolic arterial blood pressure; HR, heart rate; MAP, mean arterial blood pressure; SAP, systolic arterial blood pressure.

Figure 1 shows the time courses of the averaged cardiovascular responses to treadmill exercise. Figure 2 summarizes HR and AP responses around the onset and during the later period of exercise. HR, MAP, and DAP responses around the onset of exercise were independent of treadmill speed (ANOVA: *p* = 0.24, *p* = 0.674, and *p* = 0.898, respectively) as reported previously in animals and humans (Tsuchimochi et al., 2002 and figure 1 in Asahara et al., 2018), whereas SAP increased more (*p* = 0.022) during high‐speed (50–60 m/min) than low‐speed (20–30 m/min) exercise (ANOVA: *p* = 0.019). During the late period of treadmill exercise, HR, SAP, and MAP increased in proportion to the treadmill speed (ANOVA: *p* = 0.017, *p* < 0.001, and *p* < 0.001, respectively), whereas DAP did not differ significantly (*p* ≥ 0.072) with each treadmill speed condition.

### 3.2 Cardiac baroreflex responses to aortic occlusion during treadmill exercise at different speeds

**FIGURE 3:**
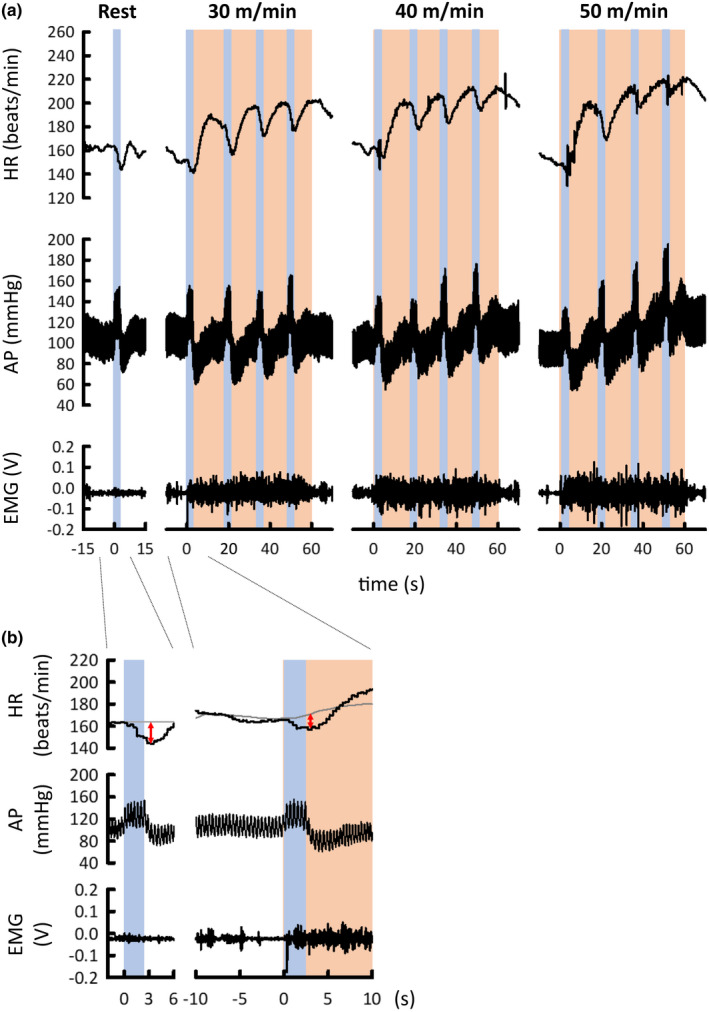
Examples of cardiac baroreflex responses to brief occlusion of the abdominal aorta at rest and during treadmill exercise at three levels of speed in a cat. (a) Baroreflex bradycardia caused by aortic occlusion appeared to be blunted at the onset of treadmill exercise, irrespective of speed. Thereafter, it recovered to the resting level during treadmill exercise at 30 and 40 m/min, while it was blunted again during the latter half of treadmill exercise at 50 m/min. (b) The inhibition of baroreflex bradycardia (two‐headed red arrows) at the exercise onset is shown at a higher chart speed. The data were taken from (a) as indicated by dotted lines. The gray line of the left side (at rest) indicates preocclusion HR, while the gray line of the right side (during the transition from rest to exercise) indicates control HR response to treadmill exercise without occlusion. Orange areas indicate periods of treadmill exercise. Light blue areas indicate periods of occlusion. AP, arterial blood pressure; EMG, electromyogram; HR, heart rate.

Figure 3 exemplifies the cardiac baroreflex responses to repeated aortic occlusion during treadmill exercise at different speeds in a cat. At rest, brief aortic occlusion caused a quasirectangular increase in AP, followed by baroreflex bradycardia (Figure 3b). The baroreflex bradycardia seemed to be blunted at the onset of treadmill exercise irrespective of the speed. After that, it was restored during exercise at low and moderate speeds, while it was blunted again during exercise at high speed (Figure 3a). The baroreflex operating point appeared to be shifted upward and/or rightward from rest to exercise, and the shift increased in proportion to treadmill speed.

**FIGURE 4:**
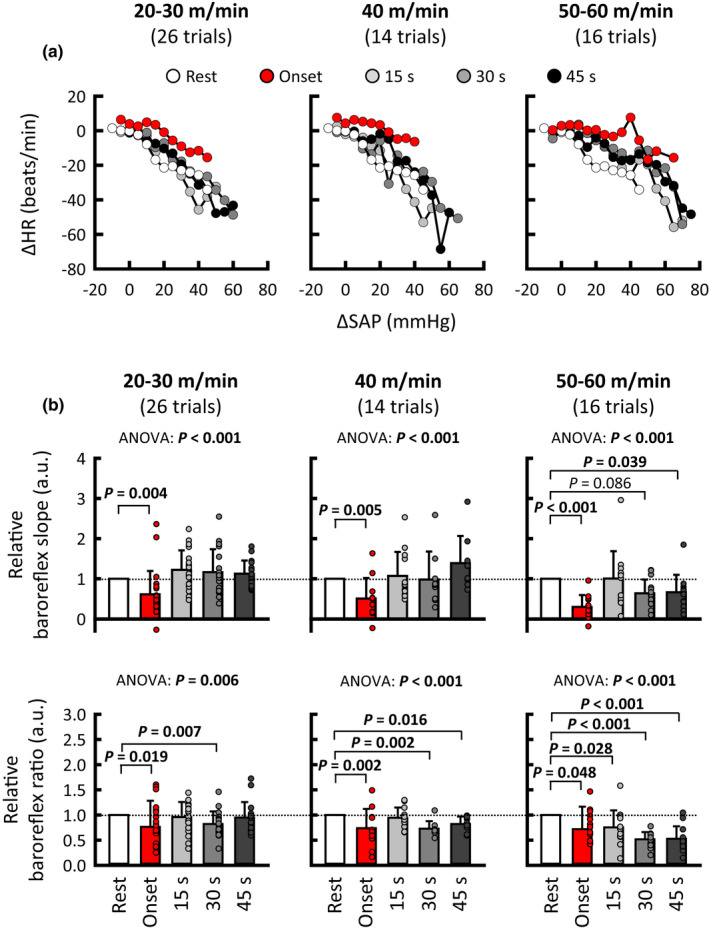
Averaged baroreflex curves between changes in SAP and HR in response to aortic occlusion (a) and the relative baroreflex slope and ratio (b) at rest and during treadmill exercise. The baroreflex ratio was calculated by dividing the net baroreflex‐induced decrease in HR by the peak increase in SAP due to aortic occlusion (detailed information was described in “Methods”). In each cat, relative baroreflex slope and ratio were calculated against the individual mean values at rest, which were taken as “1.” The baroreflex curve and slope were altered at the onset (red) of treadmill exercise, irrespective of speed. As exercise proceeded (light gray → gray → dark gray), the baroreflex curve and slope were restored to the resting level (white) in the cases of low (20–30 m/min) and moderate speed (40 m/min), whereas the inhibition of baroreflex slope occurred again at 45 s of exercise at high speed (50–60 m/min). A similar blunting effect on the relative baroreflex ratio was confirmed. The cardiac baroreflex curves are shown as means without standard deviations for data visibility, while relative baroreflex slopes and ratios are shown as means ± standard deviations. Each *P* value for post hoc tests is described when *p* < 0.1. *p* < 0.05 are in bold. HR, heart rate; SAP, systolic arterial blood pressure.

**FIGURE 5:**
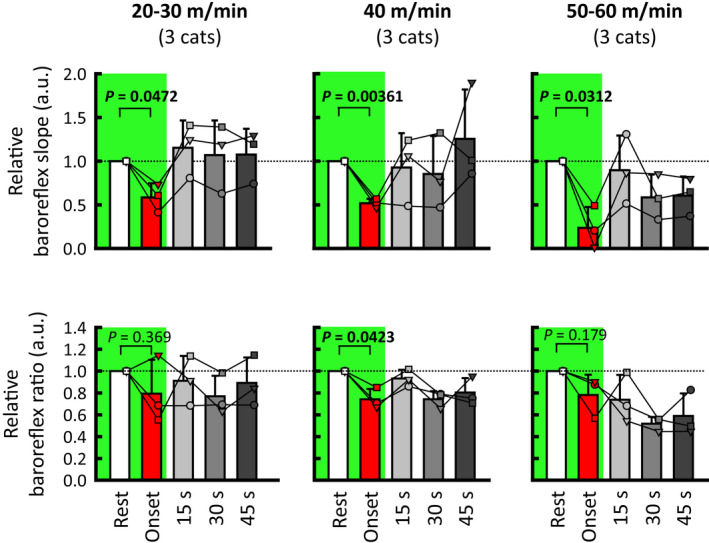
Averaged relative baroreflex slope and ratio during treadmill exercise for the three cats. In each cat, relative baroreflex slope and ratio were calculated against the individual mean values at rest, which were taken as “1.” The averaged relative baroreflex slope and ratio for the three cats were examined, and the values at rest and at the onset of exercise were compared by using a paired *t* test (as indicated by green areas). The relative baroreflex slope was significantly blunted at the onset of treadmill exercise, irrespective of the speed. The inhibitory effect on relative baroreflex ratio at the onset of exercise was significant only at 40 m/min due to the small number of cats (20–30 m/min, *p* = 0.369; 40 m/min, *p* = 0.0423; 50–60 m/min, *p* = 0.179). Each *p* value is described. *p* < 0.05 are in bold.

The baroreflex HR responses were sorted according to every 5 mmHg change in SAP, and stimulus–response curves between changes in SAP and HR are summarized in Figure 4. The maximal slopes of the stimulus–response curves, considered baroreflex sensitivity (Matsukawa et al., 2012; Matsukawa et al., 2013; Matsukawa et al., 2015; Matsukawa et al., 2016), were compared between rest and exercise. A significant main effect of exercise time on the relative baroreflex slope was observed, irrespective of the exercise speed (ANOVA: *p* ≤ 0.001). The post hoc tests demonstrated that the relative baroreflex slope was significantly (*p* ≤ 0.005) decreased to 50% or less at the onset of treadmill exercise. The same blunting effect on relative baroreflex slope was observed (*p* ≤ 0.0472) in the averaged values for the three cats (Figure 5). The decreased slope was restored at 15 s from exercise onset, irrespective of treadmill speed. The relative baroreflex slope was blunted again (*p* = 0.039) at 45 s of treadmill exercise at high speed. Given that HR increases (i.e., HR is not stationary) at the onset of exercise, the ΔHR_net_ in response to aortic occlusion and baroreflex ratio were calculated. Figure 4b shows that the relative baroreflex ratio (ΔHR_net_/ΔSAP) was changed by exercise in a similar way to the relative baroreflex slope (ANOVA: *p* ≤ 0.006). A similar blunting effect on the relative baroreflex ratio was observed in the averaged values for the three cats (Figure 5).

### 3.3 Moment‐to‐moment modulation of cardiac baroreflex during exercise

**FIGURE 6:**
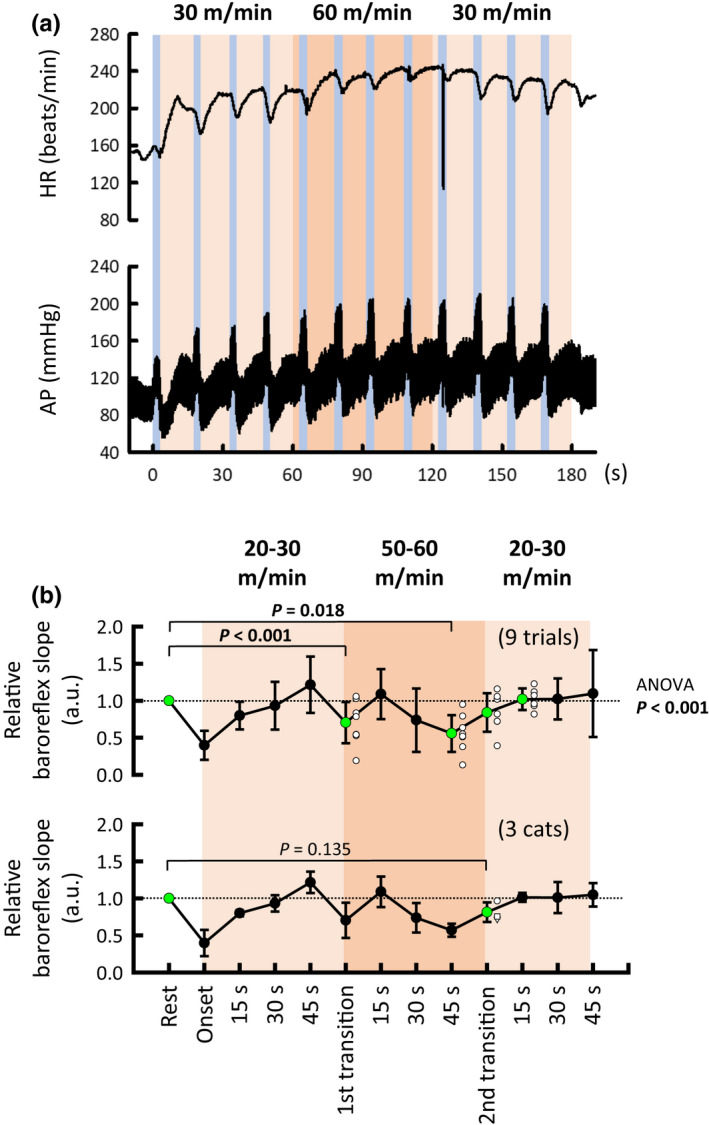
Moment‐to‐moment modulation of cardiac baroreflex during treadmill exercise with convex changes in speed. (a) An example of cardiac baroreflex responses to brief aortic occlusion during treadmill exercise with convex changes in speed (30 → 60 → 30 m/min) in a cat. In this case, arrhythmia occurred around 120 s of exercise. (b) Averaged baroreflex slope during the stepwise treadmill exercise summarized in nine trials (upper panel) and three cats (lower panel). In each cat, the relative baroreflex slope was calculated against the individual mean value at rest, which was taken as “1.” Values indicated by the green circle were statistically compared against the resting value to test our hypothesis. The relative baroreflex slope was blunted (*p* < 0.001) during the speed transition from low to high. On the other hand, such inhibition of baroreflex was restored immediately during the speed transition from high to low (*p* = 0.277 in trials; *p* = 0.135 in cats), and then the restored slope was maintained until the end of the exercise. Each *p* value for post hoc tests is described when *p* < 0.1. *p* value for the paired *t* test is described irrespective of *p* value. *p* < 0.05 are in bold. AP, arterial blood pressure; HR, heart rate.

**FIGURE 7:**
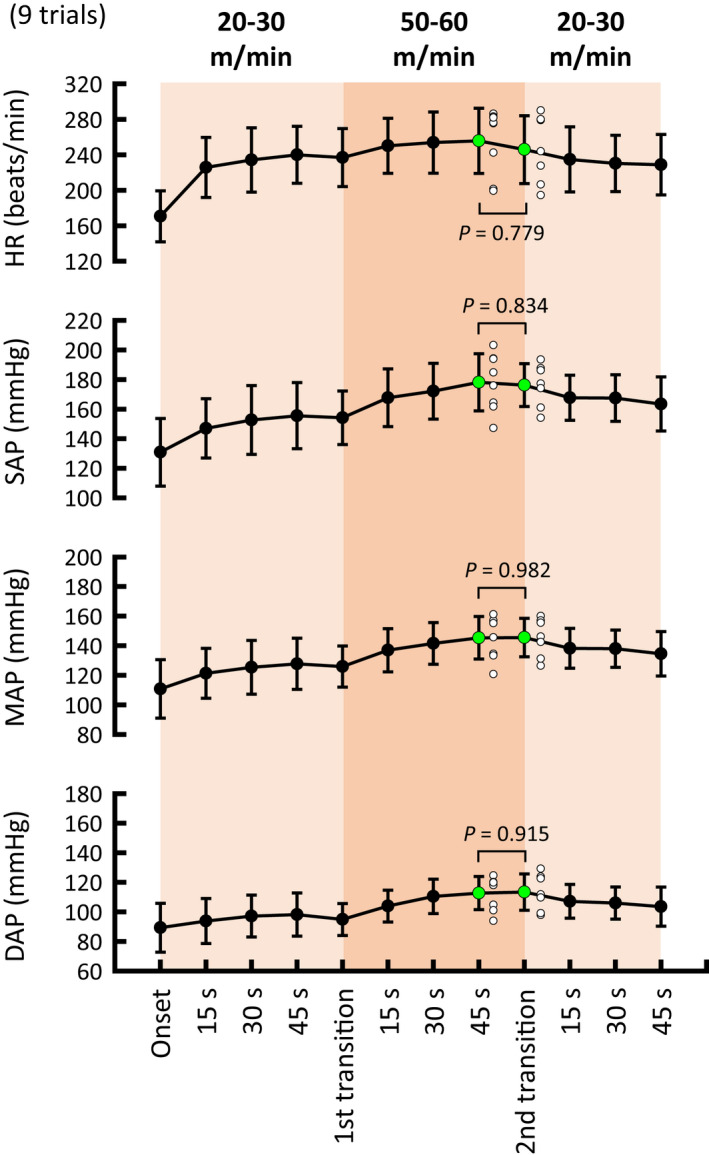
Preocclusion values of HR and AP during treadmill exercise with convex changes in speed. HR and AP values changed depending on the treadmill speed. It is noteworthy that the values of cardiovascular variables were similar (*p* ≥ 0.779) at 45 s of exercise at high speed and during the second speed transition (from high to low). Each *p* value for post hoc tests is described. AP, arterial blood pressure; DAP, diastolic arterial blood pressure; HR, heart rate; MAP, mean arterial blood pressure; SAP, systolic arterial blood pressure.

To examine the possibility that baroreflex bradycardia is blunted during transitions of treadmill speed from low to high (i.e., increasing exercise intensity), but not from high to low (i.e., decreasing exercise intensity), aortic occlusion was repeatedly performed during treadmill exercise with convex changes in speed (Figure 6). The relative baroreflex slope was blunted (ANOVA: *p* < 0.001) during the speed transition from low to high (*p* < 0.001) and at 45 s of high‐speed exercise (*p* = 0.018). Thereafter, the blunted baroreflex slope immediately returned (*p* = 0.277) to the resting level as soon as treadmill speed decreased from high to low, whereas the increases in AP and HR (i.e., preocclusion values) at 45 s of high‐speed exercise were sustained (*p* ≥ 0.779) during the speed transition from high to low (Figure 7). The restoration of the relative baroreflex slope was also confirmed (*p* = 0.135) in the averaged values for the three cats (Figure 6b).

## 4 DISCUSSION

Accumulated evidence from previous animal and human studies (Barbosa et al., 2016; Bristow et al., 1969; Ebert, 1986; Fisher et al., 2007; Komine et al., 2003; Matsukawa et al., 2012; Matsukawa et al., 2013; Matsukawa et al., 2015; Matsukawa et al., 2016; Murata et al., 2004) posed the important question of whether the cardiac component of the arterial baroreflex is inhibited even at the onset of low‐intensity exercise. The present study, using brief occlusion of the abdominal aorta, examined the effect of relatively low‐intensity exercise on cardiac baroreflex sensitivity and the presumed mechanisms responsible for baroreflex modulation in conscious cats. The major findings of this study are that (1) the cardiac baroreflex sensitivity was blunted at the onset of treadmill walking, irrespective of the speed; (2) the cardiac baroreflex sensitivity was restored at 15 s from exercise onset, irrespective of the speed, and in the case of high‐speed exercise, the blunting of cardiac baroreflex sensitivity occurred again at 45 s; (3) during stepwise exercise, the cardiac baroreflex sensitivity was blunted during the speed transition from low to high and at 45 s of high‐speed exercise; and (4) the blunted cardiac baroreflex sensitivity during high‐speed exercise immediately returned to the resting level during the speed transition from high to low. Collectively, these findings suggest that cardiac baroreflex is modulated moment‐to‐moment during exercise in association with motor intention (i.e., exercise onset) and effort (i.e., the transition of exercise intensity).

### 4.1 Dynamic modulation of cardiac baroreflex during exercise

To better understand the cardiac baroreflex function during exercise, it is important to examine the cardiac component of the entire arterial baroreflex, instead of the carotid sinus baroreflex alone, because of the superior role of the aortic baroreflex in HR control in both animals and humans (Ferguson et al., 1985; Ishii et al., 2015; Pickering et al., 2008; Shi et al., 1993; Smith et al., 2001). Since it is impossible to examine moment‐to‐moment changes in the cardiac component of the arterial baroreflex during exercise in humans, we utilized brief aortic occlusion for activation of the arterial baroreflex in conscious cats. As a result, attenuated sensitivity of the cardiac baroreflex was observed at the onset of treadmill walking, irrespective of speed (Figures 4 and 5). Even after taking the increase in HR at exercise onset into consideration, the intensity‐independent reduction of cardiac baroreflex ratio was evident. Therefore, the cardiac baroreflex function is suppressed even at the onset of low‐intensity exercise, probably due to inhibition of the cardiac component of aortic baroreflex (Komine et al., 2003; Matsukawa et al., 2012; Matsukawa et al., 2013). As exercise intensity increases, the suppression of cardiac baroreflex function at the onset of exercise would become more evident by the additional inhibition of the cardiac component of the carotid sinus baroreflex (Barbosa et al., 2016; Fisher et al., 2007).

Whether the cardiac baroreflex function is maintained (Bevegård & Shepherd, 1966; Ogoh et al., 2003; Papelier et al., 1994; Potts et al., 1993) or blunted (Cunningham et al., 1972; Kim et al., 2012; Pickering et al., 1972; Staessen et al., 1987) during exercise is still controversial. A source of this controversy is methodological differences in assessing the baroreflex (i.e., spontaneous AP changes and pharmacological, electrical, and mechanical stimulation of aortic and/or carotid sinus baroreflex) (Raven et al., 1997). Using aortic occlusion to cause a mechanically evoked rise in AP, we demonstrated that the cardiac baroreflex sensitivity returned to the resting level at 15 s of treadmill exercise, irrespective of the speed (Figure 4). The cardiac baroreflex sensitivity remained similar to the resting level during the latter half of exercise at low and moderate speeds, whereas it was blunted again at high speed. The present results at the onset of and during exercise suggest that the cardiac baroreflex sensitivity is modulated moment‐to‐moment during exercise, in association with motor intention (i.e., at the onset of exercise) and sense of effort (e.g., continuing high‐speed exercise). The degree of motor effort may be another source of the conflicting results for cardiac baroreflex function during exercise.

### 4.2 Mechanisms responsible for moment‐to‐moment modulation of cardiac baroreflex during exercise

The central command has been suggested as a major contributor to the moment‐to‐moment modulation of cardiac baroreflex sensitivity during exercise (Ebert, 1986; Komine et al., 2003; Matsukawa et al., 2012; Matsukawa et al., 2013; Matsukawa et al., 2015; Matsukawa et al., 2016; Murata et al., 2004). If this is true, the central command would blunt cardiac baroreflex sensitivity during increasing motor effort, such as increasing treadmill speed. Conversely, decreasing motor effort, such as that which occurs during decreasing treadmill speed, would immediately weaken or stop such feedforward modulation of the cardiac baroreflex. However, the central command‐like dynamic modulation of cardiac baroreflex has not been validated.

Another possible mechanism of baroreflex modulation is exercise pressor reflex via group III (predominately mechanosensitive) and group IV (predominately metabosensitive) afferent fibers in skeletal muscles. Both afferents are stimulated from the onset of evoked locomotion (Adreani et al., 1997), while some group IV fibers are stimulated by metabolic accumulation (Hayes et al., 2006; Kaufman et al., 1983). Our results show that the reflex via group III fibers (muscle mechanoreflex) is not likely to be responsible for the modulation mechanism because cardiac baroreflex was not blunted constantly in spite of presumed rhythmic activation of group III fibers throughout the exercise (Figure 4). This notion is supported by previous findings that muscle stretch had no effect on cardiac baroreflex sensitivity (Matsukawa et al., 2012; Murata et al., 2004). In contrast, the reflex via group IV fibers (muscle metaboreflex) might modulate cardiac baroreflex as metabolites would accumulate during exercise. Therefore, it is reasonable that such baroreflex modulation by muscle metaboreflex might be maintained even during decreasing treadmill speed, due to the time it takes to wash out metabolites adequately.

Based on the above rationale, we examined whether cardiac baroreflex sensitivity is modulated during transitions of treadmill speed from low to high and from high to low. We found that the cardiac baroreflex sensitivity was blunted during increasing treadmill speed and at 45 s of high‐speed exercise, but it immediately returned to the resting level during decreasing treadmill speed (Figure 6), suggesting feedforward modulation of the cardiac baroreflex. One may speculate that metabolites may be washed out during the speed transition from high to low, and thereby the baroreflex modulation by exercise pressor reflex might be restored. However, the recovery of cardiac baroreflex sensitivity in the transition phase occurred with sustained increases in AP and HR as much as those observed at 45 s of high‐speed exercise (Figure 7). Thus, exercise pressor reflex (especially muscle metaboreflex) would still operate to increase AP and HR during the transition but fail to blunt the cardiac baroreflex. To support this suggestion, most studies (Matsukawa et al., 2012; Murata et al., 2004; Potts & Mitchell, 1998; Smith et al., 2003) did not find that cardiac baroreflex was inhibited by the exercise pressor reflex, except for one study that used an arterially perfused, decerebrate rat preparation (Potts et al., 2003). Accordingly, these findings prompt us to suggest that central command is a key mechanism for the moment‐to‐moment modulation of the cardiac baroreflex during exercise. A caveat is, however, that the effect of central command on the cardiac baroreflex was not experimentally isolated (or distinguished) from that of exercise pressor reflex in this study. More direct evidence rather than reasoning will be needed to assure our suggestion in future research.

### 4.3 Presumed central mechanism for cardiac baroreflex modulation during exercise

The cardiac baroreflex modulation must occur along the central baroreflex pathway within the brain stem, but not via afferent inputs from baroreceptors, because both the aortic and the carotid sinus baroreceptor activities showed the same stimulus–response property during spontaneous motor activity as that observed at rest in decerebrate cats (Matsukawa et al., 2014). The central baroreflex pathway travels from the nucleus tractus solitarius (NTS), which receives input from baroreceptors, directly to the cardiac vagal motoneurons in the nucleus ambiguus (NA) and dorsal motor nucleus of the vagus (DMV) or indirectly to the sympathetic premotor neurons in the rostral ventrolateral medulla via GABAergic interneurons in the caudal ventrolateral medulla (Dampney, 2016; Wang et al., 2001). Considering this anatomical pathway and the major role of cardiac parasympathetic nerve activity in baroreflex bradycardia (Kollai & Koizumi, 1979; Ogoh et al., 2005; Pickering et al., 1972; Schwartz et al., 1973), central baroreflex modulation via the cardiac parasympathetic pathway would occur in the NTS and/or NA and DMV. Indeed, some neurons within the NTS, NA, and DMV receive neural inputs from higher brain centers (Dampney, 1994; Inui & Nosaka, 1993; Mussa & Verberne, 2013; Nosaka et al., 1993; van der Kooy et al., 1984). Moreover, since the cardiac component of aortic baroreflex was selectively modulated at the onset of spontaneous, fictive motor activity (Matsukawa et al., 2012; Matsukawa et al., 2013), a central command may postsynaptically influence the aortic baroreceptor‐sensitive second‐order neurons within the NTS (Matsukawa et al., 2016). As exercise intensity increases (i.e., the central command is augmented), baroreceptor‐sensitive neurons of the carotid sinus along the central baroreflex pathway may be modulated additionally by the central command, because of inhibition of the cardiac component of carotid sinus baroreflex occurred as exercise intensity increased (Barbosa et al., 2016; Fisher et al., 2007).

### 4.4 Experimental limitations

This study has several potential limitations. First of all, a small number of cats were recruited for this study because some cats appeared to be unwilling to exercise on the treadmill. Instead of a large sample, we utilized the number of trials for examining our hypothesis as was done in previous animal studies (Matsukawa et al., 2012; Matsukawa et al., 2013; Matsukawa et al., 2014; Matsukawa et al., 2015; Matsukawa et al., 2016). To confirm the statistical results, averaged data for the three cats were also analyzed (Figures 5 and 6). Second, no depressor intervention was conducted during exercise. Thus, the complete baroreflex curve and related parameters (such as gain, centering point, and upper and lower plateaus of the curve) were not examined. One may speculate that the present findings of inhibition of the cardiac baroreflex during treadmill exercise might have resulted from the shift in the operating point away from the centering point to the threshold region (i.e., upper plateau portion) of the reflex function curve (Ogoh et al., 2005). However, this concern seems to have little impact on our findings because, in this study, the occlusion‐induced pressor response (Δ ≈ 40–60 mmHg) from the operating point apparently covers the range of baroreflex HR responses (Miki et al., 2003; Papelier et al., 1994; Potts et al., 1993; Smith et al., 2001). Third, baroreflex bradycardia was mechanically evoked by brief occlusion of the abdominal aorta. This intervention could not control the magnitude of the pressor response at rest and during treadmill exercise. Finally, we regarded the treadmill speed as exercise intensity as used previously (Tsuchimochi et al., 2002), but the relative intensity of exercise was unknown. However, since the treadmill speeds used in this study caused slow to high speed of walking, but not gallop, as reported previously (Hoffer et al., 1987; Kaya et al., 2003), we expect that the intensities of 1‐min treadmill exercise would be low to moderate. In addition, exercise intensity would differ with each treadmill speed because treadmill exercise increased SAP in proportion to the speed (Figure 2), and the treadmill speeds used in this study evoke graded recruitment of anterior thigh motoneurons (Hoffer et al., 1987).

## 5 CONCLUSION

We have demonstrated for the first time that the sensitivity of the cardiac baroreflex is blunted even at the onset of low‐intensity exercise and during the transition of exercise intensity from low to high, but not from high to low. This means that cardiac baroreflex modulation occurs in association with motor intention and effort. Our rationale and present results prompt to suggest that the moment‐to‐moment modulation of cardiac baroreflex originates from central command rather than exercise pressor reflex. Future studies need to directly verify the mechanism responsible for the baroreflex modulation.

### 5.1 Physiological and clinical significance

The transition from low to high motor activity (i.e., from rest to exercise or from low‐ to high‐intensity exercise) requires the heart to supply enough blood to contract skeletal muscles and the brain. In such situations, the central command must operate to inhibit the cardiac baroreflex function, as well as to decrease cardiac parasympathetic outflow and increase cardiac sympathetic outflow, so as to increase cardiac output regardless of AP responses. Such cardiovascular regulation by central command might be exaggerated in some pathological conditions such as myocardial infarction (Koba et al., 2006) and hypertension (Liang et al., 2016). Intriguingly, augmented cardiac acceleration at the onset of exercise is a strong and independent predictor of cardiac death and nonfatal myocardial infarction in patients with coronary artery disease (Falcone et al., 2005). Accordingly, understanding the mechanisms of cardiovascular regulation during the transition from low‐ to high‐intensity activities would help to optimize the effectiveness of risk management and treatment based on the mechanisms in cardiac patients.

## AUTHOR CONTRIBUTIONS

K.I. and K.M. conceived and designed the research; All authors acquired the data. K.I. and M.I. analyzed the data. K.I., M.I., and K.M. interpreted the data. K.I. drafted the manuscript. All authors revised the manuscript, provided intellectual feedback, and approved the final manuscript.

## FUNDING INFORMATION

This study was supported by Grants‐in‐Aid for Challenging Exploratory Research: Grants‐in‐Aid for Scientific Research (B) (25560262,15H03061) from the Japan Society for the Promotion of Science.

## CONFLICT OF INTEREST

No conflicts of interest, financial, or otherwise are declared by the authors.
